# The Non-inferiority of Human Papillomavirus Vaccine Immunogenicity Among Women Over 26 Years: A Systematic Review

**DOI:** 10.7759/cureus.65157

**Published:** 2024-07-22

**Authors:** Omniah Andijani, Sara Alsalhi

**Affiliations:** 1 Preventive Medicine, Saudi Ministry of Health, Jeddah, SAU

**Keywords:** clinical trials, women, human papillomavirus vaccine, hpv, s: immunogenicity

## Abstract

Objective: Demonstrate the immunogenicity of the human papillomavirus (HPV) vaccine for the older age group (above 26 years) to prevent HPV infection with high-risk types and argue for extending vaccination recommendations for the older age group.

Methods: Two authors searched PubMed, Embase, and the Cochrane Library from inception to December 2023 to collect information on clinical trials of HPV vaccine immunogenicity. The database search strategy used a combination of subject terms and free terms. Two authors first identified studies by reading the title, abstract, and full texts and, subsequently, based on the inclusion criteria. Studies eligible to be included are the clinical trials using one of the following types of HPV vaccines: 2vHPV, 4vHPV, and 9vHPV, and measuring the immunogenicity by the geometric mean concentration or titer (GMC/T) and seroconversion rate (SCR) among healthy women aged 9 to 55 years who had never received a prophylactic HPV vaccine, known serostatus for HPV, non-immunocompetence, or non-pregnant.

Results: This review included nine articles, seven RCTs, and two open-labeled studies.

Conclusion: In summary, we have demonstrated that the immunogenicity of the HPV vaccines is non-inferior in the older age group. Even though the GMT declines with age, the SCR is similar in all age groups regardless of the serostatus. The immunogenicity of the bivalent vaccine is superior to that of the quadrivalent vaccine for the older age group. Additionally, the vaccine is more efficient in women under the age of 26, but older women will benefit from it.

## Introduction and background

Rationale

Human papillomavirus (HPV) is a viral reproductive tract infection that causes various conditions in men and women. HPV is the most common sexually transmitted infection (STI). The incidence of HPV infection peaks soon after the initiation of sexual activity. Therefore, sexually active women are at risk of developing this infection. It is transmitted through intimate skin-to-skin contact with an infected person, resulting in genital warts, cervical, anal, penile, vaginal, vulvar, and oropharyngeal cancers. The majority of HPV infections are asymptomatic and resolve spontaneously. However, persistent infection with HPV may result in precancerous lesions that may progress to cancer. There are over 200 types of HPV strains, which vary in their oncogenic potentials. The non-oncogenic strains (LR-HPV) are 6 and 11, which cause genital warts. The oncogenic strains (HR-HPV) are 16, 18, 31, 33, 35, 39, 45, 51, 52, 56, 58, and 59. HPV 16 and 18 are responsible for 70% of cervical cancer [1]. Worldwide, cervical cancer is the fourth most frequent cancer in women, and nearly all cases are associated with HPV infections. Globally, there were an estimated 604,000 new cases of cervical cancer and over 300,000 related deaths in 2020 [1]. Additionally, it is estimated that HPV infections are associated with approximately 124,000 cases of anal, oropharyngeal, penile, vaginal, and vulvar cancers [2]. Fortunately, HPV infection is considered a preventable disease through vaccination. Thus, long-term protection against HPV is required to reduce the prevalence and burden of this infection and its sequelae. In 2018, the WHO pledged to eliminate cervical cancer globally as a part of the WHO's Global Strategy. HPV prophylactic vaccination is a foundational pillar of this strategy, which aims to fully vaccinate 90% of girls by age 15 against HPV, which will be achieved by 2023. In this regard, the first generations of HPV prophylactic vaccination were approved by the United States Food and Drug Administration (US FDA) in 2006 [3]. "The World Health Organization (WHO) estimates that the HPV vaccine will save more than 4 million women's lives in low- and middle-income countries over the next decade" [4]. The United States Advisory Committee on Immunization Practices (US ACIP) recommended routine vaccination for females to start at the age of 9 years through the age of 26 years [5]. The HPV prophylactic vaccines are licensed in over 130 countries. The three prophylactic licensed vaccines for use are 9-valent (9vHPV, Gardasil 9, Merk), quadrivalent (4vHPV, Gardasil, Merk), and bivalent (2vHPV, Cervarix, GlaxoSmithKline) [6-8]. The bivalent HPV targets HPVs 16 and 18. Additionally, they are targeted by all three types of vaccines. The quadrivalent HPV targets HPV 6, 11, 16, 18, and genital warts. The nine-valent HPV targets HPV 16, 18, and five other additional strains of HPV, 31, 35, 45, 52, and 58, which are associated with 20% of HPV-associated cervical cancers. HPV vaccines have a strong immunogenic response and can elicit a robust systemic immune response through the production of antibodies by activation of naïve B-cells and T-cells. The T-helper cells stimulate naïve B-cells to produce long-lived plasma cells (LLPC) and memory B-cells, which are essential for long-term protection [3]. RCTs found that vaccinated individuals show higher antibody levels than unvaccinated individuals [9]. Three types of assays are used to measure the HPV antibodies post-vaccination: pseudovirus-based neutralization assays (PBNA), competitive Luminex immunoassays (cLIA), and enzyme-linked immunoassays (ELISA) [3]. Therefore, there are variations between studies measuring post-vaccination immunogenicity due to the different assay types used. To overcome this issue, the International Union (IU) is recommended to facilitate this comparability. The geometric mean concentration or titer (GMC/T) or the percentage of seropositivity is commonly used to measure the immunogenicity of the vaccine. The WHO and US ACIP recommended vaccination initiation in early adolescence (between ages 9 and 14) because studies have demonstrated that early vaccine administration results in greater immunogenicity and long-lasting protection. Nonetheless, young women have the highest chance of contracting HPV; women over 25 are still susceptible [3]. Recent studies demonstrate non-inferior immunogenicity among adult women aged 27-45 years and the durable effectiveness of the HPV vaccine among this age group. In this review, we will demonstrate the immunogenicity of the HPV vaccine for the older age group (above 26 years) to prevent HPV infection with high-risk types and argue for extending vaccination recommendations for the older age group. For an improved rate of HPV vaccination, misconceptions regarding vaccinations exclusively affecting sexually active women need to be corrected.

## Review

Methods

Search Strategy

From the time the database was established to December 2023, we located the published studies for HPV vaccine clinical trials in PubMed, Cochrane Library, and Embase. The search was conducted using the following keywords: immunogenicity, HPV vaccine, and human papillomavirus vaccine, in addition to filtration by sex (female) and study design (clinical trials) (Appendix 1). Studies eligible to be included are clinical trials using one of the following types of HPV vaccines: 2vHPV, 4vHPV, and 9vHPV and measuring the immunogenicity by the GMC/T and seroconversion rate (SCR) among healthy women aged 9 to 55 years who had never received a prophylactic HPV vaccine, known serostatus for HPV, non-immunocompetence, or non-pregnant. Studies written in languages other than English, incomplete works such as conference abstracts, several publications from a single randomized clinical trial (only the most recent ones were included), phase I clinical trials, and duplicate studies were all excluded.

Data Extraction

Two authors (OA and SA) independently screened the included articles and extracted data using the Review Manager software (RevMan version 5.4). Core study information, methodological information, and outcome measures were extracted, including the following: main author name, study design, year of publication, country, aim, vaccine type, age group, sample size, intervention group, control group, follow-up duration, and lastly, measured outcome.

Risk of Bias Assessment

We utilized two tools for assessing the risk of bias. The RoB2.0 tool* has been used to assess RCTs, and the ROBINS-i tool* is used to evaluate open-labeled studies. Studies may be rated as having low, moderate, or some concerns and a high risk of bias in each domain. The highest-rated domain was used to determine the overall risk of bias; for instance, if a study has at least one domain evaluated as "high risk of bias," the overall risk is deemed high. Based on quality, no studies were disqualified from the analysis. The Robvis Visualization Tool* was used to visualize reports for risk assessment.

Results

Article Selection Process

**Figure 1:**
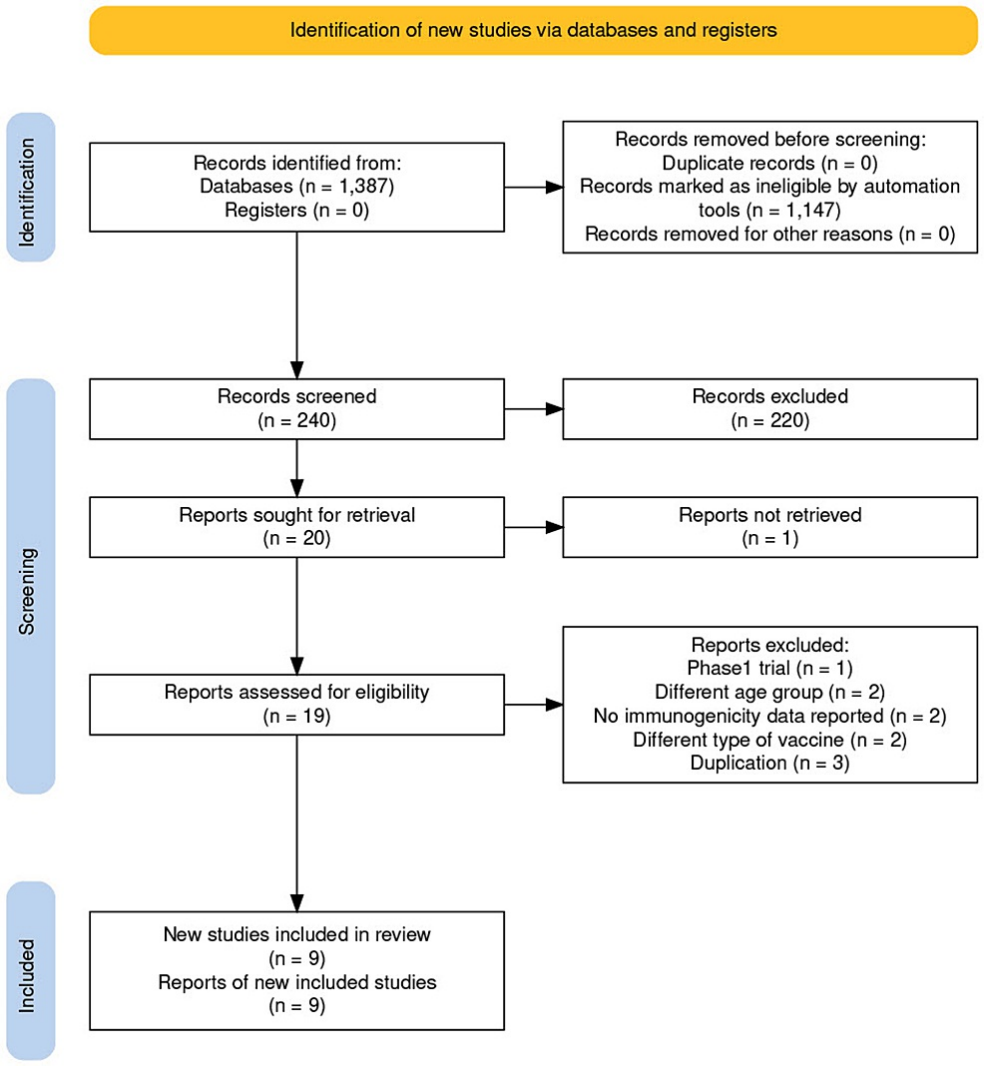
Prisma flow chart

Following the initial search, 1,387 publications were found. The automation tool excluded 1,147 publications based on the inclusion criteria. Two researchers then independently examined the 240 included articles, looking through the titles and abstracts before reading the whole text. A third reviewer was consulted if there was any disagreement. Nine studies were eventually selected. Figure 1 depicts the screening procedure for the particular article.

Characteristics of the Included Studies

Table 1 presents the basic characteristics of the included studies. All nine studies evaluated the immunogenicity with the safety of a specific HPV vaccine (bivalent, quadrivalent, or nine-valent); two of them were open-labeled trials, and the rest of the studies were RCTs. From all of the nine articles, four studies evaluated the immunogenicity of recipients of bivalent HPV vaccines against placebo vaccines (study no. 1, 2, 4, and 6). One study measured the immunogenicity of the quadrivalent vaccine against a placebo (study no. 3). Study no. (5) evaluated the immunogenicity against another HPV vaccine (bivalent vs. quadrivalent). The last three studies, which are study nos. (7, 8, and 9), had different types of controls. Both studies no. (7) and no. (8) were uncontrolled open-labeled trials, which means there were no control groups. In study no. (7), the comparison of the bivalent HPV vaccine was between age group stratifications (15-25 years, 26-45 years, and 46-55 years). In study no. (8), they compared the nine-valent human papillomavirus vaccine between women 16-26 years of age and 27-45 years of age. Lastly, study no. (9), had a different type of RCT control: recombined quadrivalent and nine-valent vaccines against the Gardasil vaccine (quadrivalent vaccine) as the control group vaccine. Each vaccine group was stratified into 20-26 years, 27-35 years, and 36-45 years. The age group stratification evaluated varied widely in all the other included studies. The range of the age groups studied was 9 to 55 years, varying from the shortest one year to the longest ten years. Three follow-up studies (no. 3, 5, and 7) reported long-term immunogenicity with follow-ups up to six years, five years, and ten years, respectively. One study (no. 6) measured the long-term immunogenicity of the bivalent HPV vaccine. In all studies, the immunogenicity outcomes were measured using GMT and SCR.

**Table 1:** Characteristics of the included studies

Studies	Study design	Publication year	Vaccine studied	Protocol number	Assessed outcomes	Participants	Intervention group	Control group	Follow-up years	Outcomes of immunogenicity
Bhatla et al. [10]	RCT	2010	Bivalent vs. placebo	NCT00344032	Immunogenicity and safety	Women aged 18–35 years	176 received bivalent vaccine	178 received placebo	1.6 years	GMT and SCR
Ngan et al. [11]	RCT	2010	Bivalent vs. placebo	NCT00306241	Immunogenicity and safety	Women aged 18–35 years	150 received bivalent vaccine	150 received placebo	1.3 years	GMT and SCR
Luna et al. [12]	RCT	2013	Quadrivalent vs. placebo	NCT00090220	Safety, immunogenicity, and effectiveness	Women aged 24-45 years	1910 received quadrivalent vaccine	1907 received placebo	2.2,6 years	GMT and SCR
Zhu et al. [13]	RCT	2014	Bivalent vs. placebo	NCT00996125 NCT01277042	Immunogenicity and safety	Study HPV-058 women aged 9 to 45 years. Study HPV-069 women aged 26 to 45.	374-606 received bivalent vaccine	376-606 received placebo	1 year	GMT and SCR
Einstein et al. [14]	RCT	2014	Bivalent vs. Quadrivalent	NCT00423046	Long-term immunogenicity and safety	Women aged 18–45 years	553 received bivalent vaccine	553 received quadrivalent vaccine	2,4,5 years	GMT and SCR
Wheeler et al. [15]	RCT	2016	Bivalent vs. placebo	NCT00294047	Efficacy, safety, and immunogenicity	Women older than 25 years	2209 received bivalent vaccine	2198 received placebo	7 years	GMT and SCR
Schwarz et al. [16]	Open-label trial	2017	Bivalent in women 26–45 and 46–55 years of age vs. 15-25	NCT00196937 NCT00947115	Immunogenicity and safety	Women aged 15–55 years	666 received bivalent vaccine (base study) and 451 completed the 10-year assessment	No control group	1,6,10 years	GMT and SCR
Joura et al. [17]	Open-label trial	2021	Nine-valent	NCT03158220	Immunogenicity and safety	Women 27-45 versus 16-26 years of age	570 women aged 16-26 years	642 women aged 27-45 years	1.2 years	GMT and SCR
Shu et al. [18]	RCT	2022	Nine-valent and Quadrivalent vs. placebo	4vHPV: NCT03085381 9vHPV: NCT03676101	Immunogenicity and safety	Women aged 20-45 years	560 women received quadrivalent vaccine compared with 560 received placebo	560 women received nine-valent vaccine compared with 560 received placebo	1.3 years	GMT and SCR

The Risk of Bias Assessment

**Figure 2:**
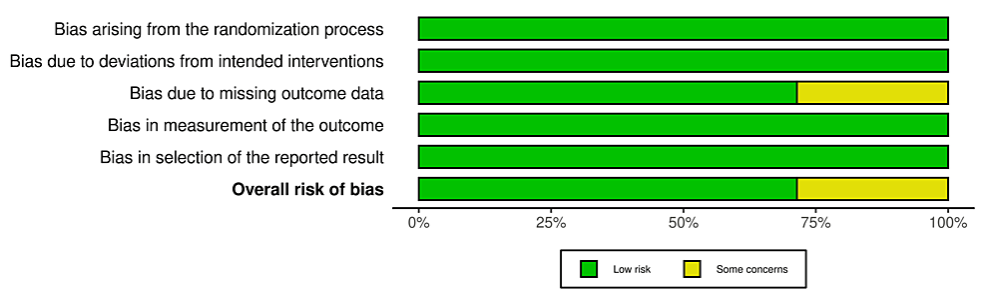
RoB2.0

**Figure 3:**
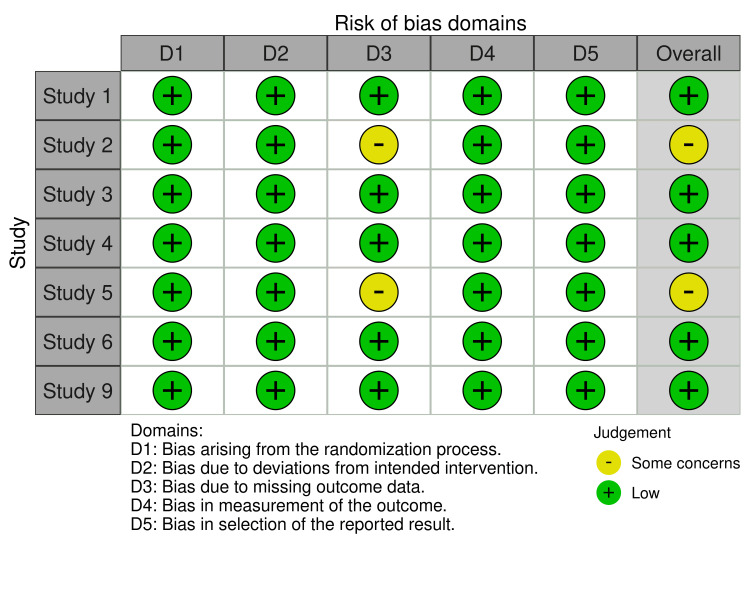
RoB2.0

**Figure 4:**
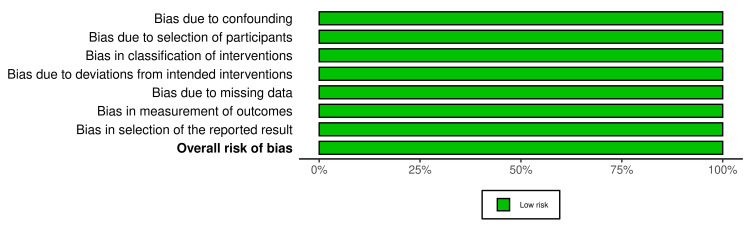
ROBINS-i

**Figure 5:**
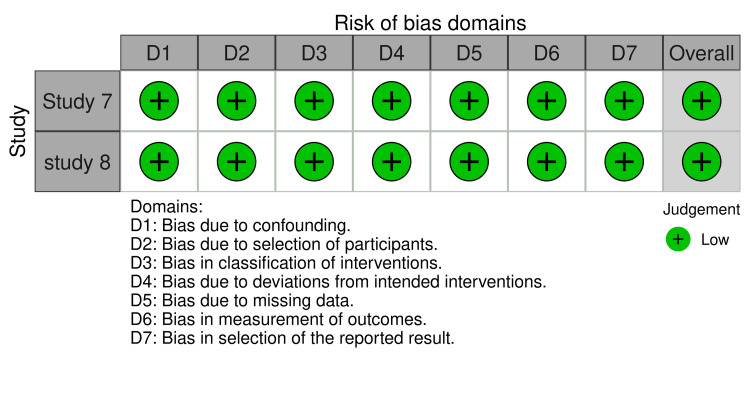
ROBINS-i

This systematic review included seven studies from RCTs and two open-labeled trials (Figures 2-3). Regarding the RCT studies, most have a low risk of bias, except for two studies (study no. 2 and 5), which have some bias concerns. Regarding selection bias, all RCTs (n = 7) were randomized trials, describing the method of sequence generation and detailing the unpredictability of random allocation of subjects. To maintain a low risk of bias, all trials were blinded and unlikely to break; for bias in the measurement of the outcomes, all RCTs had explicit blindness for outcome measurement and were unlikely to break. Five of the seven RCTs had complete results and no missing outcome data except for two studies concerned about how they dealt with the lost participants; the studies did not mention per-protocol (PP) analysis or intention-to-treat (ITT) analysis. All studies had no risk of reporting bias as they all reported completed information about participants' data (Figures 4-5). All open-labeled studies (n = 2) reported a low risk of bias. Both studies included methods of controlling the confounding factors, like stratification and matching between participants. The overall risk of bias in both studies was low.

Immunogenicity of HPV Vaccine

As natural infection does not induce reliable protection, the HPV vaccine can potentially prevent cervical cancer by inducing greater antibody titers [10]. HPV vaccines have a strong immunogenic response and can elicit a systemic immune response through the production of antibodies [19] and the activation of naïve B-cells and T-cells [3]. The T-helper cells stimulate naïve B-cells to produce LLPC and memory B-cells, which are essential to sustain high levels of neutralizing antibodies (nAb) for long-term protection [3]. The neutralizing antibodies, IgG and IgA, are assumed to be the protection mediators following vaccination. IgG titers, nAbs, and memory B-cells can monitor the immune response. The age of vaccination is important as it affects the maintenance of the immune response but not the induction of memory B-cells. The predicted prolonged immune response decreases with age, probably due to a lower initial antibody response [16]. Previous studies demonstrate that nAb remained detectable up to 7-12 years after vaccination [20-21]. Moreover, RCTs found that HPV antibody levels peak 100-fold in vaccinated individuals compared to naturally infected individuals [9]. Some RCTs comparing different vaccine types showed that the bivalent vaccine induces a higher antibody response than the quadrivalent vaccine [1]. In contrast, the nine-valent vaccine responds similarly to the quadrivalent vaccine [22].

Seroconversion is defined as the development of antibodies against HPV in the blood serum caused by either infection or immunization. The SCR for HPV is defined as the HPV-specific antibody concentration above the cut-off point and the proportion of seropositive participants [23]. Seronegative means the absence of detectable levels of antibodies. Systemic immunization with L1 VLP vaccines produces high serum antibody concentrations at least 50-1000 times those measured in natural infections, and virtually all vaccines seroconvert, in contrast to natural infections, which have a slow and weak humoral immune response, and only 50-70% of individuals seroconvert [24]. Moreover, compared to an older age, above 25, the long-term seropositivity rate (SPR) seems to be higher when the vaccine is given at a younger age [16].

Three types of assays are used to evaluate the HPV antibodies post-vaccination: PBNA, cLIA, and ELISA [3]. The PBNA measures the biological activity, whereas the cLIA measures the neutralizing activity, and the ELISA detects all antibodies regardless of their neutralization [25]. The WHO suggests that PBNA is the standard reference method for assessing HPV vaccine-produced antibodies; however, this method is expensive and time-consuming [3]. In comparison, cLIA and ELISA are fast and produce high throughput [3]. Due to the difficult comparability and variations between studies on these three assays, in addition to the unavailability of official guidelines on methods for determining the cutoffs, standardized measurements have been established using the International Unit (IU) [3]. The standardized measurement for the immunogenicity of the HPV vaccines includes the GMC/T and the percentage of seropositives (i.e., the proportion of study participants with an antibody level above a certain cut-off level) [3]. Both provide information on the long-term performance of the vaccine regarding antibody production. GMC/T levels reached by a natural infection provide a standard value for evaluating antibody levels post-vaccination [16].

Efficacy of HPV Vaccine

Numerous randomized controlled trials (RCTs) have shown the high efficacy of HPV vaccines against cervical infection and lesions that protect up to 98% [26]. A large meta-analysis on the population-level impact of HPV vaccination was conducted in nine high-income countries four years after HPV vaccine introduction. This meta-analysis showed an 83% reduction in the prevalence of HR-HPV, HPV-16/18, among young girls aged 13-19 years compared to pre- and post-vaccination implementation eight years after the implementation [27].

Bivalent (2vHPV) Vaccine

The bivalent vaccine (2vHPV, Cervarix®), manufactured by GlaxoSmithKline Biologicals, was the first licensed HPV vaccine in 2006. It targets the most important high-risk HPV types, 16 and 18, which are responsible for approximately 70% of cervical cancer cases [28]. This HPV-16/18 prophylactic vaccine consists of virus-like particles (VLPs), which resemble the L1 protein of HPV. All vaccines contain aluminum salt as an adjuvant; however, the HPV-16/18 vaccine uses the proprietary immunostimulatory adjuvant system 04 (AS04), which contains both aluminum salt and monophosphoryl lipid A (MPL) to stimulate the innate immune response [13]. The immunogenicity induced by the AS04-adjuvanted vaccine is higher than that of other non-AS04-adjuvanted vaccines [29]. Clinical trials demonstrated that the immune response generated by the AS04-adjuvanted vaccine persists for up to at least 6.4 years [30]. By mathematical modeling, the induced immunogenicity of this type of vaccine is projected to last up to 20 years without diminishing from the plateau reached within two years of vaccination [10]. This vaccine is administered in a three-dose series (0.5 mL each) by intramuscular injection in the deltoid muscle. The second dose is recommended one to two months after the first dose, and the third dose is recommended six months after the second dose (zero, one, and six-month schedule).

Quadrivalent (4vHPV) Vaccine

Quadrivalent vaccine (4vHPV, Gardasil®), manufactured by Merck, was the second licensed HPV vaccine in 2007. It targets HPV types 11 and 6, which account for 90% of anogenital warts [12], in addition to those in 16 and 18. This prophylactic HPV-6/11/16/18 contains L1 VLPs of HPV-6/11/16/18 and is formulated with a proprietary amorphous aluminum hydroxyphosphate sulfate (AAHS) adjuvant. This vaccine is administered in a three-dose regimen (0.5 mL each), and the doses are scheduled for zero, one to two, and six months.

Nine-Valent (9vHPV) Vaccine

Nine-valent vaccine (nonavalent, 9vHPV, Gardasil9®), manufactured by Merck, was licensed in 2014 targeting HPV types 6, 11, 16, and 18, in addition to five high-risk types, which are 31, 33, 45, 52, and 58. This prophylactic HPV-6/11/16/18/31/33/45/52/58 vaccine has the potential to prevent 90% of cervical cancer and HPV-related vulvar, vaginal, and anal cancers, as well as 90% of genital warts [18]. In clinical trials, partial cross-protection was observed for HPV type 31 by bivalent and quadrivalent vaccines and for HPV type 45 by bivalent vaccines [18]. The nine-valent vaccine is similar to the quadrivalent vaccine in the adjuvant system. It consists of an AAHS adjuvant. This vaccine is given in a three-dose series according to the following schedule: zero, one to two, and six months. The doses are administered as a 0.5-mL intramuscular injection in the deltoid muscle of the non-dominant arm (Table 2).

**Table 2:** Information on the available HPV vaccine. MPLA: monophosphoryl lipid A, AAHS: amorphous aluminum hydroxyphosphate sulfate.

	Bivalent vaccine (2vHPV) Cervarix®	Quadrivalent vaccine (4vHPV) Gardasil®	Nine-valent or nonvalent vaccine (9vHPV) Gardasil9®
Year listened	2006	2007	2014
Manufacturer	GlaxoSmithKline Biologicals	Merck	Merck
Target HPV types	HPV-16/18 (HR-HPV)	HPV-6/11 (LR-HPV) HPV-16/18 (HR-HPV)	HPV-6/11 (LR-HPV) HPV-16/18/31/33/45/52/58 (HR-HPV)
Adjuvant	500 μg aluminum hydroxide, 50 μg 3-O-deacylated-4-MPLA	225 μg AAHS adjuvant	500 μg AAHS adjuvant
Schedule	0, 1, and 6 months	0, 1-2, and 6 months	0, 1-2, and 6 months

Discussion

Literature

As part of its Global Strategy, WHO pledged in 2018 to end cervical cancer as a worldwide health issue. The cornerstone of this approach is HPV preventive vaccination, with the intention of having 90% of girls by the age of 15 fully immunized against HPV by 2023. In 2006, the first HPV vaccination was approved by the US FDA. As of 2022, girls' routine vaccination programs in 125 countries included the HPV vaccine. For maximum effect, the WHO recommends focusing on young adolescent girls before sexual activity. Studies of newly acquired HPV infection demonstrate that the incidence of infection peaks soon after first sexual activity in most populations [31]. In a study among college women, the cumulative incidence of infection was 28.5% one year after sexual debut and increased up to 50% by the third year [32]. Since the prevalence of HPV infection starts after the initiation of sexual activity, vaccination among adolescent girls is preferred. However, covering the entire life period of sexual activity will result in optimal benefits [3]. While young women are more likely to contract HPV, women over 25 years of age are also susceptible, especially when it comes to new sexual partners [33-34].

The literature reveals that the HPV vaccine's immunogenicity reaches its optimal benefit if administered to adolescent girls; therefore, this review explores the immunogenicity of older women to include this age group in the vaccination recommendations. This systematic review identified nine clinical trials that evaluated the immunogenicity of different types of HPV vaccine among women stratified by age, mainly above 26 years and below 26 years, by measuring the GMT and SCR.

Risk of HPV Infection and Sexual Activity

Starting with study no. 1 [10], which included 330 Indian women aged 18-35 years vaccinated with the bivalent HPV vaccine, the GMT level at month seven post-vaccination (one month after the last dose of the vaccine) among initially seropositive women was high, which supports that the vaccine-induced immunity can protect sexually active and those who have previously been exposed to natural infection, as well as HPV-naïve women. Additionally, study no. 2 [11], which included 300 Chinese women aged 18-35 years, included 24% initially seropositive and vaccinated with the bivalent HPV vaccine. Evidence showed that the immune response among them is similar to that of seronegative women, which indicates that prior exposure to HPV infection does not affect the immune response generated by the HPV vaccine. Furthermore, study no. 3 [12], which included 3,817 women aged 24-45 years from multiple countries vaccinated with the quadrivalent HPV vaccine, demonstrated that sexually active women who are above the age of 26 have potential benefits from the vaccination. Since those adult women are at risk of acquiring new HPV infections and related sequelae, they should have the opportunity to choose to be vaccinated on an individual basis.

Vaccine Immunogenicity

Bivalent vaccine: Study no. 4 [13] included 1,962 Chinese women aged 9-45 years (stratified into 9-17 years, 18-25 years, and 26-45 years) vaccinated with the bivalent HPV vaccine, and this is the only study in this review that included very young age. The study showed that the GMT for anti-HPV-16 and anti-HPV-18 were higher in those aged 9-17 years by two- to three-fold compared with those aged 18-25 years, concluding that there is a decrease in GMTs with increasing age. Moreover, this study demonstrated that seropositivity increases with age due to cumulative HPV exposure, consistent with the previously reported data in the literature.

Bivalent and Quadrivalent Vaccines

Study no. 5 [14] is a head-to-head study comparing the long-term immunogenicity and safety of the bivalent and quadrivalent HPV vaccines five years post-vaccination. The study was conducted among 1,106 healthy women aged 18-45 years and stratified into age groups: 18-26 years, 27-35 years, and 36-45 years. The findings of this study showed the GMTs of nAb for anti-HPV-16 and -18 from the bivalent vaccine in women aged 18-26 years were 7.8 and 12.1-fold higher, respectively, than those from the quadrivalent vaccine in women aged 27-35. The GMTs of nAb for anti-HPV-16 and -18 were 5.6- and 13-fold higher, respectively. Lastly, in women aged 36-45, the GMTs of nAb were 2.3- and 7.8-fold higher, respectively. Thus, the GMTs of nAbs for HPV-16 and -18 induced by the bivalent HPV vaccine were higher than those elicited by the quadrivalent HPV vaccine in all age groups. The nAb elicited by the bivalent HPV vaccine remains longer and above the levels associated with natural infection than those induced by the quadrivalent HPV vaccine. Furthermore, the GMTs of the nAb peak at month 7, then decline and reach a plateau from month 18 onward. This plateau level induced by the bivalent HPV vaccine was significantly higher than the level caused by the quadrivalent HPV vaccine among all age groups.

Additionally, the plateau levels of the bivalent HPV vaccine across all age strata remained several-fold higher than the level associated with natural infection. This indicates the inferiority of the quadrivalent HPV vaccine for the older age group. However, there was a noticeable decrease in SCR for anti-HPV-18 below 80% around year five post-vaccination among women aged 36-45. This aligns with study no. 3 and implies a decline over time in the proportion of the subjects seropositive for HPV-18 by the cLIA assay. Meanwhile, in month 72, the SPR for HPV-18 measured by the cLIA assay was 45% among all age groups, whereas for HPV-6/11/16, the SPR was maintained at 90% or higher. However, the SPR for HPV-18 measured by the total IgG assay exceeded 80%, which is much higher than the cLIA SPR due to the particular features of each assay. Moreover, women aged 24-34 years expressed a higher response to all HPV types by the cLIA assay compared with women aged 35-45 years of age.

Long-Term Immunogenicity in Adult Women

Bivalent vaccine: Study no. 6 [15] (VIVIANE study) is the most extended phase 3 HPV vaccine follow-up efficacy trial. This study included only women over 25 years (stratified into 26-35 years, 36-45 years, and older than 45) vaccinated with the bivalent HPV vaccine. They followed up participants for HPV infection with HPV DNA testing every six months and Pap cytology every 12 months. This is the only study in our review that assessed the efficacy and immunogenicity outcomes of HPV vaccination. The findings from this study show that the immune response was sustained across all age groups, with only a slight decline in antibody titers between the four-year and seven-year analyses, which aligns with the findings from other studies in this review. The seven-year analysis of VIVIANE confirms the efficacy of the bivalent HPV vaccine in preventing infection and mild cytological abnormalities associated with the bivalent HPV vaccine in adult women, with cross-protection against HPV 31 and HPV 45. These findings support extending vaccination to women older than 25 years. While adolescent vaccination remains a priority, adult women can still benefit. Strategies like "vaccinate and screen" or "screen and vaccinate" could improve the cost-effectiveness of adult vaccination programs.

Study no. 7 [16] (open-label trial) is this review's most extended follow-up trial. The study evaluated the 10-year immune persistence and long-term safety of the bivalent vaccine in women aged 15-55 years. This study demonstrated the persistent immunogenicity of the bivalent HPV vaccine in women aged 15-55, with an acceptable safety profile for at least ten years following the initial vaccination. Thus, the catch-up programs would benefit women in the older age group who are not targeted by immunization. This conclusion is consistent with the VIVIANE study.

*Nine-valent vaccine: *The second open-label trial (study no. 8 [17]) was conducted among 1,212 women aged 16-45 years in multiple countries to assess the immunogenicity and safety of the nine-valent HPV vaccine. This study supports the findings in the study (no. 3) that adult women remain at risk of acquiring new HPV infections. This trial showed that 86% of women were not infected with any of the HPV types (6/11/16/18/31/33/45/52/59) targeted by the nine-valent vaccine. Additionally, over 99% in both age groups (16-26 years and 27-45 years) were seroconverted for the nine strains of HPV. In terms of antibody response, this trial showed that older women aged 27-45 years were non-inferior to those younger women aged 16-26 years. Additionally, there was a trend of declining GMTs with advancing age, which aligns with the results from earlier research. The quadrivalent vaccine demonstrated good efficacy across all age groups despite decreasing GMTs with age, suggesting the clinical relevance of decreased immunogenicity is minimal.

*Quadrivalent and nine-valent vaccine: *The last RCT (no. 9 [18]) was conducted among 1,680 Chinese women aged 20-45 years to assess the safety and immunogenicity of the recombinant 4- and 9-valent against Gardasil. The study concludes that these two vaccines are highly immunogenic and well-tolerated among Chinese women aged 20-45. Moreover, 99% of the participants were seroconverted by month 7 for HPV-6/11/16/18 in all stratified age groups (20-26 years, 27-35 years, and 36-45 years). As observed in the previous studies, this RCT concludes that the GMT levels for all HPV types are inversely correlated with age.

Limitations

The literature needs more clinical trials in our Middle Eastern population for the results and conclusions from this review to be more generalizable.

Recommendation 

Our recommendation from this review is to extend the vaccination to women aged above 26, proposing the use of a bivalent vaccine. We recommend using two strategies to improve the cost-effectiveness of vaccination in older women: the vaccinate and screen strategy and the screen and vaccinate strategy [35].

## Conclusions

In summary, we have demonstrated that the immunogenicity of the HPV vaccines is non-inferior in the older age group even though the GMT declines with age, the SCR is similar in all age groups regardless of the serostatus. The immunogenicity of the bivalent vaccine is superior to the quadrivalent vaccine for the older age group. Furthermore, the vaccine efficacy is higher in women below 26 years old, but older women will benefit from the vaccination.

## Appendices

Search keywords

(((((immunogenicity) AND (HPV)) OR (immunogenicity)) AND (Human papilloma virus vaccine)) OR (immunogenicity)) AND (Human papillomavirus vaccine)

Tools

1. PRISMA flow diagram tool:

PRISMA Flow Diagram (shinyapps.io)

2. ROBINS-i tool:

https://www.riskofbias.info/welcome/home/current-version-of-robins-i/robins-i-tool-2016

3. RoB2.0:

20190822_RoB_2.0_guidance_parallel_trial.pdf - Google Drive

4. visualization tool for risk of bias:

https://mcguinlu.shinyapps.io/robvis/
